# Turner Syndrome Associated With Refractory Seizures and Intellectual Disability: A Case Study

**DOI:** 10.7759/cureus.11364

**Published:** 2020-11-06

**Authors:** Manami Akasaka, Atsushi Kamei, Jun Ito, Kotaro Oyama

**Affiliations:** 1 Pediatrics, Iwate Medical University, Morioka, JPN

**Keywords:** unclassified epilepsy, neurodevelopmental disorder, refractory seizure, intellectual disability, fluorescence in situ hybridization, g-band analysis, mosaicism, spikes shifting foci, lamotrigine and valproic acid combination therapy, turner syndrome

## Abstract

Turner syndrome (TS) is the most frequent sex abnormality in women. The physical features include short stature, webbing of the neck, and gonadal dysgenesis. Typically, patients with Turner syndrome exhibit no intellectual disability, and a few cases of TS have been associated with epilepsy. Herein, we present a case of TS with intractable epilepsy. The patient presented with global developmental delay at the age of two and karyotyping revealed mosaicism [45, X/46, X del (X) (q21.1)]. At the age of seven, she had generalized tonic epilepsy as well as several focal-onset seizures. She developed daily seizures, which were refractory to several antiepileptic drugs. Interictal electroencephalography (EEG) revealed multifocal spikes, and ictal EEG revealed shifting foci. She visited our hospital at the age of 13. Her peripheral white blood cells G-band and fluorescence in situ hybridization (FISH) method chromosome with cheek swab examinations revealed 45, X. Her peripheral white blood cell mosaic pattern may have disappeared over time or become indetectable. We treated her with clobazam, and then lamotrigine and valproic acid combination therapy, which resulted in a reduction in the frequency of seizures by approximately 50%. Epilepsy and intellectual disability in this case may be due to the mosaic deletion at Xq21.1. Further analysis of similar cases may provide valuable information for effective therapeutic strategies.

## Introduction

Turner syndrome (TS) is one of the most common sex chromosome disorders involving the loss of a part or all X-chromosome functions. TS occurs in approximately 1 in 2000 to 2500 live female births. Physical features of girls with TS include short stature, webbing of the neck, gonadal dysgenesis, congenital heart diseases, and urinary system malformations [1]. TS is generally associated with normal intelligence, but neurological complications, such as autism spectrum disorder, attention deficit hyperactivity disorder, visual-spatial cognitive impairment, and psychiatric diseases are more frequently found in female patients with TS than in females without TS [1]. The X-chromosome contains several genes involved in neuronal morphogenesis, neuroanatomical abnormalities, and epilepsy [2]. However, epilepsy is unusual in TS, with a morbidity rate of approximately 2-3% [3,4]. TS with epilepsy is frequently associated with structural brain abnormalities and mosaicism karyotype [5-13]. As there are few reports on TS with epilepsy, the relationship between epilepsy and the X-chromosome in TS is unclear. This case highlights the unusual association of this syndrome with intractable seizures, moderate developmental delay, and an apparent karyotyping inconsistency. This case will be described in detail and compared and reviewed in the context of previous case reports. 

## Case presentation

The patient was the first child of healthy non-consanguineous Japanese parents. She was born at 40 weeks gestation with a birth weight of 2558 g. At the age of two, she presented with a moderate developmental delay. She visited another hospital, and her karyotype was revealed as 45, X/46, X del (X) (q21.1). At the age of six, her height was significantly shorter than her peers, and she received growth hormone replacement therapy. Her intellectual development was also moderately impaired (intelligence quotient was 44 with the Tanaka-Binet intelligence test). At the age of seven, she began to have generalized tonic epilepsy during sleep. Interictal electroencephalography (EEG) exhibited multifocal spikes, and she was diagnosed with epilepsy. She was treated with valproic acid (maximum dosage of 15 mg/kg/day for two years), which was effective for only two years. Valproic acid (VPA) was ineffective after those two years and then generalized seizures recurred, and in addition, she began to have several focal-onset seizures, hyperkinetic facial seizures (right or left side deviation of the head and eyes), and autonomic seizures (pale face and drooling). Her seizures occurred over 10 times a day and more often during sleep. She was treated with carbamazepine (maximum dosage of 10 mg/kg/day for four years) and levetiracetam (maximum dosage of 60 mg/kg/day for two years), but they were ineffective. At the age of 13, she was admitted to our hospital. She exhibited physical features of webbing of the neck, delayed secondary sexual characteristics, and congenital left kidney malformation. No cardiovascular abnormality was detected. Her blood test results and serum chemistry were normal. Brain MRI revealed no abnormal findings. Both her peripheral white blood cells G-band and fluorescence in situ hybridization (FISH) method chromosome with cheek swab examinations revealed 100% 45, X. She had focal to bilateral tonic-clonic seizures, focal facial motor seizures, and autonomic seizures during the night. The ictal EEG exhibited abnormal epileptic discharges in the right and left hemispheres. In addition, abnormal EEG spikes revealed shifting foci from the right to the left hemisphere. We also observed generalized spikes during generalized tonic seizures. The addition of clonazepam (maximum dosage of 0.02 mg/kg/day for only two months, after which it was discontinued) led to drowsiness and was ineffective. Her seizures remained poorly controlled, and she suffered from seizures more than 10 times a day. Lamotrigine (LTG) (2 mg/kg/day) was added to her treatment, and she underwent pre-surgical evaluation at the age of 15; however, surgery was not indicated at this time. Clobazam (dosage of 0.2 mg/kg/day) was added, but her seizures did not improve, moreover, it led to drowsiness, and then we reduced the dosage (0.1 mg/kg/day). We decided to treat her with combination therapy of LTG (2 mg/kg/day) and VPA (dosage was 8 mg/kg/day) because previous reviews have suggested a good response in controlling refractory seizures with combination therapy [14,15]. Her seizure frequency reduced by approximately 50%.

## Discussion

TS is rarely associated with severe intellectual developmental disorders and epilepsy. In our case, the patient revealed mosaic karyotype as 45, X/46, X del (X) (q21.1) at the age of two, moderate intellectual developmental delay, and generalized tonic epilepsy at the age of seven. Two years later, she began to have several focal-onset seizures refractory to anticonvulsant therapies. Interictal EEG showed multifocal spikes, and ictal EEG revealed shifting foci from the right hemispheres to the left. We diagnosed her with unclassified epilepsy characterized by focal ictal migration.

An English literature survey from 1990 until 2020 was done using the keywords “Turner syndrome” and “epilepsy” in the PubMed database and Japan Medical Abstracts Society, and only nine case reports have appeared (Table 1) [5-13]. In six of these cases, the karyotype analysis showed mosaicism, although the mosaic karyotyping 45, X/46, X del (X) (q21.1) had never reported in the present case. Seven patients displayed abnormalities on brain MRI include focal cortical dysplasia, periventricular heterotopia, polymicrogyria, lissencephaly, mild brain atrophy, and asymmetrical ventricles. Most of the patients were treated with multiple anticonvulsants. However, the outcome in 90% of these cases was poor, which is consistent with our case. LTG/VPA combination therapy, as reviewed by Moeller et al., showed a positive outcome in treating up to two-thirds of patients with refractory epilepsy [14]. Grisotto et al. showed a retrospective analysis of 37 children with refractory epilepsies taking LTG/VPA. Efficacy of seizure control was considered satisfactory if there was a reduction in seizures >50% or total control. They revealed a satisfactory treatment response was obtained in 65% of children with this combination therapy [15]. In contrast to a previous case study using LTG/VPA combination, our patient’s seizures were reduced significantly by about 50% [12]. Therefore, this combination therapy should be tried in cases of intractable epilepsy with TS.

**Table 1:** Published case studies on Turner syndrome associated with refractory seizures GTCS：generalized tonic clonic seizure; FBTCS：focal to bilateral tonic clonic seizure; FIAS: focal impaired awareness seizure; GAS：generalized absence seizure; FMS：focal motor seizure; VPA：valproic acid; OXZ：oxcarbazepine; CZP：clonazepam; CBZ：carbamazepine; PHT：phenytoin; PB：phenobarbital; ZNS：zonisamide; TPM：topiramate; LTG：lamotrigine; ND：not described

Case	Karyotype	Age at onset	Type of seizure	Brain CT/MRI	Treatment	Outcome	Reference No.
1	45, X	1 year	focal onset seizure, FBTCS	bilateral perisylvian hypoplasia	ketotic diet, VPA, OXZ	poor	[5]
2	45, X /46, XX	10 years	FIAS	lissencephaly	ND	poor	[6]
3	45, X	8 months	rub epilepsy	focal cortical dysplasia	VPA, TPM	poor	[7]
4	45, X/46, XX	12 years	FIAS, automatisms, FBTCS	bilateral frontal polymicrogyria	VPA, CBZ, CZP	good	[8]
5	45, X	8 years	focal onset seizure, automatisms, GTCS	mild brain atrophy	Chinese herb, CZP, TPM, CBZ	poor	[9]
6	45, X/46, XX /47, XXX	11 years	GTCS	asymmetrical ventricle	CBZ, VPA, Chinese herb	poor	[10]
7	45, X/46, XX	30 years	GTCS, automatisms	normal	PB, LTG, VPA, TPM, OXZ	poor	[11]
8	46, X, del (Xp22.33)	10 years	GAS	normal	VPA, LTG	poor	[12]
9	45, X/47, XXX	18 years	GTCS	lissencephaly	PB, PHT, VPA, CZP	poor	[13]

Typically, TS cases show no intellectual disability. In this case, the initial karyotype G-band analysis at the age of two showed mosaicism with a deletion at Xq21.1. X-linked intellectual disability is the most extensively studied genetic phenomenon involving the human central nervous system. Seizures accompany intellectual disability in almost half of the syndromes caused by mutation of genes on the X-chromosome [2,16]. However, a deletion at Xq21.1 is poorly described in the literature and only a few cases are reported of male patients with complex phenotypes including intellectual disability. About 50% of the carrier females display almost or completed skewed X inactivation, silencing the abnormal X resulting in a relatively benign phenotype [17]. The Xq21.1 deleted region contains 14 genes, three genes (BRWD3, TBX22, and POU3F4) have been previously reported mutated in defined disorders. Mutation in the BRWD3 gene is known as X-linked intellectual disability. Epilepsy and intellectual disability in our case may be due to the mosaic deletion at Xq21.1. In our case, the peripheral white blood cell G-band analysis revealed mosaicism at the age of two. Her clinical symptoms, particularly, the mental retardation and intractable seizures, were suggestive of mosaicism; however, at the age of 13, it revealed 100% 45, X.

Rasouli et al. reported that in cases where conventional karyotype results do not closely match the clinical presentation, FISH analysis with interphase cells may be informative for mosaicism [18]. Therefore, we additionally performed the FISH analysis and the cheek swab examinations during the same time as the G-band analysis; however, both examinations also revealed 100% 45, X. Her peripheral white blood cell mosaic pattern may have disappeared over time or become indetectable. The timing of karyotype analysis may be important in the diagnosis of mosaicism.

 Takahashi et al. reported several cases with mixed gonadal dysgenesis (MGD). They revealed that conventional karyotype analysis of peripheral lymphocytes by G-band has several limitations in the diagnosis and clinical evaluation of MGD. First, the detection of mosaicism depends on in vivo or in vitro selection against one of the cell lines, i.e., the number of the cells analyzed is one of the critical factors and natural selection of the normal cell line in lymphocytes may occur both during the prenatal period and after delivery. Second, karyotype analysis with peripheral lymphocytes may not reflect that in the organs. The FISH analysis may be more useful for detecting low-frequency mosaicism [19]. However, we were unable to examine other tissues or skin fibroblasts for a deeper analysis because the patient’s family did not provide consent. In the future, we should analyze karyotype with fibroblast or repeat FISH to gain gene information more precisely.

## Conclusions

The majority of case reports of epilepsy associated with TS revealed mosaic pattern were intractable. Further analysis of similar cases is required to gain a better understanding of the mechanisms involved and for designing effective therapeutic strategies.
